# Severity of frailty using modified Thai frailty index, social factors, and prediction of mortality among community-dwelling older adults in a middle-income country

**DOI:** 10.3389/fmed.2022.1060990

**Published:** 2022-12-09

**Authors:** Ekkaphop Morkphrom, Varalak Srinonprasert, Unchana Sura-amonrattana, Arunotai Siriussawakul, Supawadee Sainimnuan, Rinrada Preedachitkun, Wichai Aekplakorn

**Affiliations:** ^1^Division of Geriatric Medicine, Department of Medicine, Faculty of Medicine Siriraj Hospital, Mahidol University, Bangkok, Thailand; ^2^Siriraj Geriatric Internal Medicine Research Group, Research Department, Faculty of Medicine Siriraj Hospital, Mahidol University, Bangkok, Thailand; ^3^Integrated Perioperative Geriatric Excellent Research Center, Faculty of Medicine Siriraj Hospital, Mahidol University, Bangkok, Thailand; ^4^Siriraj Health Policy Unit, Faculty of Medicine Siriraj Hospital, Mahidol University, Bangkok, Thailand; ^5^Department of Anesthesiology, Faculty of Medicine Siriraj Hospital, Mahidol University, Bangkok, Thailand; ^6^Department of Community Medicine, Faculty of Medicine Ramathibodi Hospital, Mahidol University, Bangkok, Thailand

**Keywords:** frailty, older, mortality risk, Thailand, caretaker

## Abstract

**Background:**

Frailty has been increasingly recognized as a public health problem for aging populations with significant social impact, particularly in low- and middle-income countries. We aimed to develop a modified version of the Thai Frailty Index (TFI) and explore the association between different frailty statuses, socioeconomic factors, and mortality in community-dwelling older people from a middle-income country.

**Methods:**

The data from participants aged ≥60 years in the Fourth Thai National Health Examination Survey were used to construct the 30-item TFI. Cutoff points were created based on stratum-specific likelihood ratio. TFI ≤ 0.10 was categorized as fit, 0.10–0.25 as pre-frail, 0.25–0.45 as mildly frail, and >0.45 as severely frail. The association of frailty status with mortality was examined using Cox proportional hazard models.

**Findings:**

Among 8,195 older adults with a mean age of 69.2 years, 1,284 died during the 7-year follow-up. The prevalence of frailty was 16.6%. The adjusted hazard ratio (aHR) for mortality in pre-frail was 1.76 (95% CI = 1.50–2.07), mildly frail 2.79 (95% CI = 2.33–3.35), and severely frail 6.34 (95% CI = 4.60–8.73). Having a caretaker in the same household alleviated mortality risk for severely frail participants with an aHR of 2.93 (95% CI = 1.92–4.46) compared with an aHR of 6.89 (95% CI = 3.87–12.26) among those living without a caretaker.

**Interpretation:**

The severity of frailty classified by the modified TFI can predict long-term mortality risk for community-dwelling older adults. Identification of severely frail older people to provide appropriate care might alleviate mortality risk. Our findings can inform policymakers to appropriately allocate services in a resource-limited setting.

## Introduction

Frailty has been emphasized as a public health problem worldwide not only among aging but also pre-aging populations (1, 2). Frailty is a complex multidimensional condition that leads to negative clinical outcomes including falls, delirium, disability, and mortality among older people independent of chronological age (3). Pre-frailty is an intermediate stage between robust fitness and frailty and is also associated with delirium, prolonged hospital stay, and mortality (1, 4). Recent evidence demonstrates that early intervention in frail and pre-frail people can prevent adverse clinical outcomes and possibly reverse the frailty status (5, 6).

Multiple operational criteria to identify frailty have been developed (3, 7). Two widely used and often modified models for the diagnosis of frailty are the phenotypic (8) and accumulated deficit model (9). The limitations and strengths of these and other models are debated widely (3, 7, 10, 11) and have yielded a variety of recommendations in practice guidelines (7, 10, 11).

The Thai frailty index (TFI) (12) was established in 2018 using the accumulation deficit model and validated against mortality risk in community-dwelling older people. However, applying the TFI in research and clinical practice revealed several limitations, including prolonged administration times and difficulty in performing the assessment, particularly with gait speed measurement. Measurement of walking speed might be impractical, providing that a substantial proportion of community-dwelling older adults experience a “fear of falling” (13). Moreover, a previous meta-analysis of the influence of gait on frailty revealed heterogeneity in methods used and the magnitude of effect (14). To improve the utility of the tool for everyday practice, the modified Thai frailty index (modified TFI) that does not include the measurement of gait speed was developed.

We aimed to establish the validity of the modified TFI and to investigate different cutoff values for states of frailty to better identify vulnerable populations in the community. Moreover, we aimed to explore whether social factors such as socioeconomic status (SES) and having a caretaker would affect mortality risk among community-dwelling older persons at different frailty statuses in a middle-income country.

## Materials and methods

### Data collection and measurement

The fourth Thai National Health Examination Survey (NHES-IV) is a nationally representative cross-sectional survey using multistage, stratified sampling of the Thai population. The data for this study came from the NHES-IV that was conducted in 2009. The present study extracted data from participants aged 60 years or older for analysis against mortality outcomes. The details of sampling methods are described elsewhere (15). The NHES-IV contains information on demographic, socioeconomic, and health data such as age, gender, smoking, medication, history of falls, hearing problem, dental problem, medical comorbidities, availability of caretakers, activities of daily living (ADLs), cognitive function, and quality of life (QoL). The details of data collection have been described elsewhere (12). In brief, trained research staff measured the blood pressure, gait speed, grip strength, and body mass index with standard measurement and interviewed participants for basic activities of daily living (BADLs), instrumental activities of daily living (IADLs), and QoL using the questionnaire applied in the WHO SAGE project (16). Availability of caretakers was assessed by the question “Currently, do you have a caretaker to help with your basic activities of daily living?” The definition of caretakers in our questionnaire which could be elaborated for participants was “persons who look after the older persons for activities of daily living.” The tasks they offer help with include bathing, dressing, getting in or out of bed, walking, using the toilet, and eating. Cognitive function was assessed using the Mental State Examination Thai version–2002. Diabetes and chronic kidney disease were ascertained by enquiring about the relevant history and blood examinations, while hypertension was ascertained by measuring blood pressure and reviewing medication.

The wealth index score was utilized to represent SES in the NHES IV. The data of individuals’ household items were collected and factored into the wealth index score based on the recommended method (17). Wealth quintiles were created where the lowest quintile indicated the poorest group and the highest quintile indicated the wealthiest group. Mortality data were retrieved until May 2016 from the Thailand Vital Registration System, Bureau of Registration Administration, and Ministry of Interior.

### Development of modified Thai frailty index

The modified TFI was created following a standard procedure. In brief, the variables used in the frailty index should correlate with health status; increase with age until the late-life period; and cover physical, cognitive, and mental health (18). We selected 30 variables from different domains including medical comorbidities, functional status, physical performance, and emotional health to calculate the modified TFI (19). All variables were dichotomous (0 or 1). The frailty index was calculated as the number of items defined as a deficit divided by the 30 items considered (20). In addition to removing gait speed measurement, the 30-item Mental State Examination Thai version–2002, which requires several minutes to administer, was also eliminated. We replaced the full cognitive test with three-word recall and replaced gait speed measurement with serial-seven subtraction, considering the evidence of a reliable association between these two tests (21). All variables are listed in Supplementary Table 1.

### Statistical methods

Following published studies to validate the state of frailty, cutoff values for the frailty index were based on stratum-specific likelihood ratios (SSLRs) (22). We first used a cutoff that was commonly proposed for a frailty index from previous studies (20, 23) to separate the sample into seven strata (i.e., ≤0.03, >0.03 to ≤0.10, >0.10 to ≤0.25, >0.25 to ≤0.30, >0.30 to ≤0.33, >0.33 to ≤0.45, and >0.45). All strata were analyzed for their likelihood of experiencing mortality compared with the mortality rate of the overall cohort using the following formula:


$$\text{SSLR}=\left(\text{x}1_{\text{stratum}}/{\text{n1}}_{\text{overall}}\right)/\left(\text{x}0_{\text{stratum}}/{\text{n0}}_{\text{overall}}\right)$$

where x1 _stratum_ represents the total number of participants experiencing death in a stratum; n1_overall_ is the total number of participants who died in the overall cohort; x0 _stratum_ is the total number of participants who survived in the stratum; and n0_overall_ is the total number of participants who survived in the overall cohort.

Stratum-specific likelihood ratios statistics were expected to increase with frailty index scores. We compared the difference in SSRI of each stratum with the preceding stratum and accepted the level of statistical significance at *p* < 0.05. The strata with non-significant differences were merged into a single group. After merging, we then defined each group as fit, pre-frail, mildly frail, and severely frail according to the increasing scores.

After the cutoffs were established, baseline characteristics of frailty status were compared between groups. The chi-squared test was applied for categorical data. Parametric and non-parametric tests were applied for continuous data after examining for normality of the distribution of variables. Data with non-normal distribution were reported in the median and interquartile range [IQR]. Data with normal distribution were reported in mean and standard deviation (SD). A *p*-value of 0.05 was considered statistically significant.

The associations with mortality were examined using Cox proportional hazard models. Since several comorbidities and health conditions were included in the modified TFI, only variables not included in the modified TFI were investigated in the hazard models. Age, smoking status, and wealth index were therefore explored. Stratified analysis by gender was performed considering substantial evidence of different contributing factors between genders on mortality among older persons. Additional analyses were performed to investigate the effect of having a caretaker and SES on mortality. The concordance probability of the modified TFI and the original version was calculated using the Harrell’s C index. Statistical analyses were performed using STATA 16.0 (College Station, TX Stata Corp LP).

## Results

Our study included 8,195 seniors who participated in the NHES-IV. The mean age was 69.2 years (SD = 6.8), and 50.8% were women. Over 7 years of follow-up, 1,284 died with an average death rate of 27.1 per 1,000 person-years. The baseline characteristics of included participants according to frailty status are shown in Table 1. Table 2 illustrates mortality data and SSLR for each stratum of the frailty index. Strata FI = 0.10 to ≤0.25; 0.25 to ≤0.30; and >0.45 were significantly different in the likelihood ratio for mortality. Thus, we used cutoff scores of 0.10, 0.25, and 0.45 to categorize frailty status into four groups as followed: FI 0.00 to ≤0.10 as “fit”; 0.10 to ≤0.25 as “pre-frail”; 0.25 to ≤0.45 as “mildly frail”; and >0.45 as “severely frail.” The overall prevalence of frailty in the present cohort was 16.6% (95% CI: 15.4–17.0%), of which 15.4% were mildly frail (95% CI: 14.7–16.3%), and 1.2% were severely frail (95% CI: 1.0–1.4%). The prevalence of frailty among women was 20.4% (95% CI: 15.4–17.5%) and frailty among men was 12.8% (95% CI: 11.9–14.0%). The prevalence of pre-frailty in the present study was 56.9% (95% CI: 55.8–58.0%).

**TABLE 1:** Baseline characteristics of study participants by frailty status.

Variable	Fit (*n* = 2,170)	Pre-frail (*n* = 4,662)	Mildly frail (*n* = 1,266)	Severely frail (*n* = 97)	*P*-value
Age, mean ± SD (year)	67.4 ± 19.4	69.2 ± 6.7	72.3 ± 7.6	75.7 ± 6.9	<0.001
Good overall health status, *n* (%)*	1,532 (70.6%)	1,572 (33.7%)	161 (12.7%)	2 (2.1%)	<0.001
History of smoking, *n* (%)	1,089 (50.2%)	2,442 (52.4%)	655 (51.7%)	49 (50.5%)	<0.001
Wealth index quintile, *n* (%)					<0.001
1st	341 (15.7%)	950 (20.4%)	320 (25.3%)	23 (23.7%)	
2nd	317 (14.6%)	819 (17.6%)	229 (18.1%)	23 (23.7%)	
3rd	415 (19.1%)	944 (20.2%)	289 (22.8%)	19 (19.6%)	
4th	419 (19.3%)	922 (19.8%)	215 (17.0%)	18 (18.6%)	
5th	678 (31.2%)	753 (23.6%)	213 (16.8%)	14 (14.4%)	
BMI, mean ± SD (kg/m^2^)	23.0 ± 3.6	23.3 ± 4.5	23.3 ± 4.9	22.2 ± 4.3	<0.001
Gait speed, median [IQR] (m/s)	0.74 [0.62, 0.86]	0.68 [0.56, 0.80]	0.58 [0.46, 0.69]	0.48 [0.36, 0.58]	<0.001
Hand grip, median [IQR] (kg.)	21.60 [18.50, 24.50]	19.90 [16.70, 22.79]	17.70 [14.70, 20.79]	16.10 [12.90, 18.10]	<0.001
History of falls in 6 months, *n* (%)	134 (6.2%)	497 (15.6%)	827 (29.1%)	45 (46.4%)	<0.001
Number of BADL deficit**, Median [IQR]	1 [1,2]	2 [1,2]	2 [1,2]	2 [2,4]	<0.001
Number of IADL deficit***, Median [IQR]	1 [1,1]	1 [1,2]	2 [1,3]	4 [2,5]	<0.001
Number of comorbidities, median [IQR]	0 [0, 1]	1 [0, 2]	2 [1,3]	2 [2, 3]	<0.001
Living with caretaker, *n* (%)	607 (28.1%)	1,671 (36.0%)	607 (48.2%)	64 (66.0%)	<0.001
Depressive mood, *n* (%)	51 (2.4%)	792 (17.0%)	630 (49.8%)	77 (79.4%)	<0.001
Cognitive impairment, *n* (%)	94 (4.3%)	444 (9.5%)	273 (21.6%)	35 (36.1%)	<0.001
Hypertension, *n* (%)	299 (13.8%)	1,673 (35.9%)	700 (55.3%)	64 (66.0%)	<0.001
Diabetes, *n* (%)	135 (6.2%)	882 (17.6%)	344 (27.2%)	34 (35.1%)	<0.001
Stroke, *n* (%)	14 (0.6%)	119 (2.6%)	97 (7.7%)	21 (21.6%)	<0.001
Chronic obstructive pulmonary disease, *n* (%)	10 (0.5%)	117 (2.5%)	52 (4.1%)	8 (8.2%)	<0.001
Death; per 1,000 person-years (95% CI)	15.1 (13.2–17.4)	27.2 (25.3–29.2)	44.2 (39.6–49.3)	99.3 (75.7–130.3)	<0.001

*Overall health status is self-report overall heath from the question “In general, how would you rate your health today?” (very good, good, moderate, bad, very bad).

**Basic activity of daily living (BADL) deficit means inability to do one of the following by themselves: bathing, dressing, eating, transferring from bed to chair, indoor ambulation, stair climbing, toileting, urinary continence, and fecal continence.

***Instrumental activity of daily living (IADL) deficit means inability to do one of the following by themselves: paying for bill, medication use, light housework (e.g., sweeping floor, tidying room), heavy housework (e.g., mobbing floor, carrying water bucket), public transportation, telephone use. IQR means interquartile range that represent the range from 25th percentile to 75th percentile of data.

**TABLE 2:** Stratum-specific likelihood ratio (SSLR) for experiencing seven-year mortality in seven frailty-index strata using common proposed cut off.

Frailty index	Died	Survive	SSLR	95% CI
>0.00–≤0.03	32	426	0.40	0.28 to 0.58
>0.03–≤0.10	165	1,547	0.57	0.49 to 0.67
>0.10–≤0.25*	602	3,418	0.95	0.89 to 1.01
>0.25–≤0.30*	240	888	1.45	1.28 to 1.66
>0.30–≤0.35	76	254	1.62	1.26 to 2.07
>0.35–≤0.45	122	328	2.00	1.64 to 2.44
>0.45*	47	50	5.06	3.41 to 7.50

*The stratum that significant difference in mortality comparing to the preceding stratum (*p* < 0.05).

**FIGURE 1:**
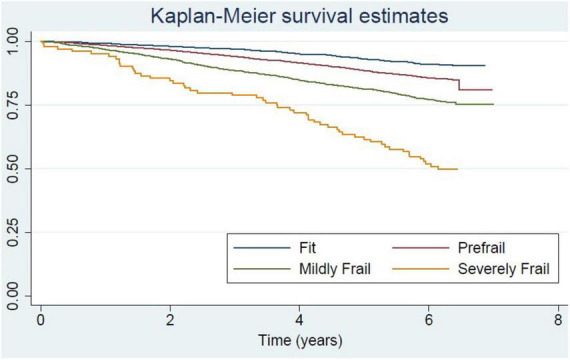
Kaplan-Meier curve for survival of community-dwelling older adults in National Health Examination Survey (NHES-IV) comparing among frailty status.

Severely frail, mildly frail, and pre-frail people were more likely to have a higher number of medical comorbidities, lower physical performance (low grip strength and low gait speed), be more dependent in activities of daily living, and have cognitive impairment commensurate with their degree of frailty (Table 1). With respect to economic status, fit older people were more likely to be in the 4th and 5th quintiles of the wealth index. This phenomenon is also apparent in the pre-frail group. In contrast, older people in frail groups were more likely to be in the lower quintiles of the wealth index. The correlation of negative health states and frailty status was consistent in both male and female cohorts. The death rate in older men was higher than in women in all groups. The death rate per 1,000 persons comparing men and women for different frailty statuses is as followed: fit 11.8 vs. 5.4; pre-frail 18.5 vs. 13.1; mildly frail 33.5 vs. 18.5; and severely frail 68.8 vs. 38.5 (Detailed information is shown in Supplementary Tables 2, 3). The Kaplan-Meier survival curve comparing each frailty status is shown in Figure 1.

Compared with fit older adults, all states of frailty including pre-frailty were independently associated with increased mortality risk after adjustment for age, smoking, and wealth index. Deaths per 1,000 person-years in fit, pre-frail, mildly frail, and severely frail were 15.1, 27.2, 44.2, and 99.3, respectively. The adjusted hazard ratio (aHR) of pre-frail, mildly frail, and severely frail compared with the fit group were 1.76 (1.50–2.07), 2.79 (2.33–3.35), and 6.34 (4.60–8.73), respectively (Table 3). Age was an independent risk factor of mortality among pre-frail, mildly frail, and severely frail persons (aHR of pre-frail = 1.07, 95% CI: 1.06 to 1.08; aHR of mildly frail = 1.05, 95% CI: 1.03 to 1.07; and aHR of severely frail = 1.05, 95% CI: 1.01 to 1.12) but not a risk factor among fit persons (adjusted HR = 1.00, 95% CI: 0.99–1.00). These associations were similar for both men and women (Tables 4, 5).

**TABLE 3:** The associations between 7 year all-cause mortality and frailty status among Thai older adults for all study participants, and compared between male and female who participated in the 4th Thai National Health Examination Survey (NHES-IV).

Frailty status	Total cohort (*n* = 8,195)		Male (*n* = 4,048)		Female (*n* = 4,147)	
			
	Mortality, % (95% CI)	Adjusted HR (95% CI)	Mortality, % (95% CI)	Adjusted HR (95% CI)	Mortality, % (95% CI)	Adjusted HR (95% CI)
Fit	9.1 (7.9–10.4)	reference	11.8 (10.0–13.7)	reference	5.4 (4.0-7.0)	reference
Pre-frail	15.7 (14.6–16.7)	1.76* (1.50–2.07)	18.5 (16.9–20.1)	1.58* (1.31–1.91)	13.1 (11.7–14.5)	2.48* (1.82–3.37)
Mildly frail	24.2 (21.8–26.6)	2.79* (2.33–3.35)	33.5 (29.3–37.8)	3.08* (2.46–3.86)	18.5 (15.8–21.4)	3.35* (2.40–4.67)
Severely frail	48.5 (38.2–58.8)	6.34* (4.60–8.73)	68.8 (50.4–83.9)	7.80* (4.96–12.26)	38.5 (26.6–51.4)	7.51* (4.61–12.23)

*P-value < 0.001; were adjusted for age, smoking, and wealth index quintiles.

**TABLE 4:** The associations between 7 year all-cause mortality and wealthy status and age among Thai older adults with different frailty status.

Variables	Fit (*n* = 2,170)	Pre-frail (*n* = 4,662)	Mildly frail (*n* = 1,266)	Severely frail (*n* = 97)
**Wealth index quintile**				
1st (least wealthy)	Reference	Reference	Reference	Reference
2nd	1.06 (0.69–1.63)	0.84 (0.68–1.06)	1.07 (0.77–1.50)	1.69 (0.74–3.82)
3rd	0.64 (0.41–1.01)	0.95 (0.77–1.18)	0.94 (0.68–1.30)	0.69 (0.27–1.81)
4th	0.60* (0.38–0.96)	0.72* (0.57–0.91)	0.84 (0.58–1.20)	0.66 (0.27–1.61)
5th	0.56* (0.37–0.86)	0.70* (0.56–0.87)	0.82 (0.57–1.19)	0.70 (0.24–2.02)
age	1.00 (0.99–1.00)	1.07* (1.06–1.08)	1.05* (1.03–1.07)	1.07* (1.01–1.12)

*P-value < 0.05; adjusted for gender and smoking.

**TABLE 5:** The associations between 7 year all-cause mortality and wealthy status and age among Thai older adults with different frailty status stratified by gender.

Variable	Men (*n* = 4,048)				Women (*n* = 4,147)			
		
	Fit (*n* = 1,255)	Pre-frail (*n* = 2,275)	Mildly frail (*n* = 486)	Severely frail (*n* = 32)	Fit (*n* = 915)	Pre-frail (*n* = 2,387)	Mildly frail (*n* = 780)	Severely frail (*n* = 65)
**Wealth index quintile**								
1st (least wealthy)	Reference	Reference	Reference	Reference	Reference	Reference	Reference	Reference
2nd	0.99 (0.60–1.62)	0.88 (0.66–1.18)	1.17 (0.74–1.84)	2.97 (0.93–9.45)	1.42 (0.61–3.30)	0.77 (0.54–1.10)	0.99 (0.61–1.61)	1.09 (0.36–3.29)
3rd	0.56* (0.33–0.95)	0.80 (0.66–1.18)	1.03 (0.66–1.61)	0.40 (0.07–2.30)	0.98 (0.41–2.33)	1.13 (0.83–1.54)	0.86 (0.53–1.38)	0.56 (0.16–1.93)
4th	0.51* (0.30–0.88)	0.70* (0.52–0.96)	0.75 (0.45–1.24)	0.57 (0.68–2.00)	1.00 (0.41–2.46)	0.72 (0.50–1.02)	1.00 (0.59–1.68)	0.60 (0.16–2.28)
5th	0.57* (0.35–0.92)	0.74* (0.55–0.99)	0.91 (0.55–1.52)	1.20 (0.31–4.68)	0.53 (0.20–1.36)	0.63 (0.43–1.36)	0.73 (0.43–1.24)	0.40 (0.07–2.15)
age	1.00 (0.99–1.01)	1.06* (1.04–1.07)	1.05* (1.03–1.07)	1.04 (0.97–1.12)	1.00 (0.99–1.01)	1.08* (1.07–1.10)	1.05* (1.03–1.08)	1.08* (1.01–1.15)

*P-value < 0.05; adjusted for smoking.

Wealth index was a protective factor for mortality among the fit and pre-frail older adults, especially in the 5th quintile, with an aHR of 0.56 (0.37–0.86) for fit and 0.70 (0.56–0.87) for the pre-frail group. The protective effect of the wealthy became insignificant among older people in both mildly frail and severely frail groups (Table 4). In subgroup analysis stratified by gender, the protective effect of wealth status differed substantially between men and women (Table 5). In men, compared to the least wealthy group, the benefit of SES on mortality was apparent in fit and pre-frail groups. The associations were stronger in the fit group, with an aHR of 0.56 (0.33–0.95), 0.51 (0.30–0.88), and 0.57 (0.35–0.92) in 3rd to 5th quintiles compared with an aHR of 0.70 (0.52–0.96) and 0.74 (0.55–0.99) in 4th and 5th quintiles for pre-frail men, respectively. In women, the protective effect of SES was not significant in all stages from fit to severely frail.

In both genders, living with a caretaker mitigated the mortality risk among pre-frail and frail older persons compared with those who lived without a caretaker (Table 6). Among severely frail older persons, the difference in mortality risk between the group living without a caretaker and with a caretaker was substantial with an aHR of 6.89 (3.87–12.26) compared to 2.93 (1.92–4.46). Moreover, gender showed a significant influence on the association with a stronger effect among men. Severely frail men living without a caretaker had an extremely high aHR of 18.78 (95% CI: 8.13 to 43.33), while severely frail men living with a caretaker had an aHR of 3.24 (95% CI: 1.82–5.76). For women living without a caretaker, the aHR was 5.97 (2.62–13.59) compared with 3.72 (1.86–7.43) for those living with a caretaker.

**TABLE 6:** The associations between 7 year all-cause mortality and frailty status comparing between different living arrangement stratified by gender.

	Total		Men		Women	
			
	History of living without caretaker (*n* = 5,207)	History of living with caretaker (*n* = 2,949)	History of living without caretaker (*n* = 2,618)	History of living with caretaker (*n* = 1,409)	History of living without caretaker (*n* = 2,589)	History of living with caretaker (*n* = 1,540)
Fit	Reference	Reference	Reference	Reference	Reference	Reference
Pre-frail	1.87* (1.52–2.29)	1.26 (0.97–1.63)	1.79* (1.40–2.28)	1.04 (0.77–1.42)	2.31* (1.57–3.39)	2.00* (1.20–3.32)
Mildly frail	2.87* (2.24–3.67)	1.64* (1.23–2.18)	3.33* (2.44–4.56)	1.78* (1.26–2.52)	3.24* (2.11–4.97)	1.95* (1.13–3.36)
Severely frail	6.89* (3.87–12.26)	2.93* (1.92–4.46)	18.78* (8.13–43.33)	3.24* (1.82–5.76)	5.97* (2.62–13.59)	3.72* (1.86–7.43)

*P-value < 0.05; adjusted for age, smoking and wealth index quintiles.

The Harrell’s C index for the modified TFI was 0.580, while the original version of the TFI was 0.565. The modified TFI provides a significantly better prediction for mortality (*p* < 0.05).

## Discussion

Using data from a nationally representative cohort, the modified version of the 30-item TFI can predict long-term mortality risk according to frailty status. The cutoff values for the frailty index were identified using the established method (20) to categorize the frailty status into pre-frail (>0.10 to ≤0.25), mildly frail (>0.25 to ≤0.45), and severely frail (>0.45). These categories of frailty classified the characteristic of the population in each group reasonably well demonstrated by the increase in deficits in domains of physical, mental, and function in a dose-response manner with the severity of frailty.

Several meta-analyses have explored the prevalence of frailty and pre-frailty among community-dwelling older adults using various tools (24–26). The prevalence of frailty in our study is similar to previous studies with similar age groups (25) but slightly lower than studies using a frailty index as the diagnostic approach (12, 24). We also observed a similar prevalence compared to the pooled result from other middle-income countries (26). The prevalence of pre-frailty was slightly higher than the pooled prevalence from all regions but fairly similar to studies from other Asian countries (24). The cutoff points of the frailty index might explain the difference in the prevalence of frailty and pre-frailty in studies using the cumulative deficit model. Most studies in one systematic review (24) applied a cutoff of 0.20 for frailty, but we used a cutoff value of 0.25 following a previous validation study with a similar approach (20).

Our study demonstrated the association between frailty index and long-term mortality risk among community-dwelling older people similar to a recent meta-analysis (27). Moreover, the phenomenon “sex-frailty paradox” identified in other studies (28), for which women had a higher prevalence of frailty but men had higher mortality at the same level of frailty index, was also apparent in our study. Several hypotheses including biological, behavioral, and social factors have been described in this phenomenon (29). Although many studies have focused on the differences in the severity of comorbidities, (30) the evidence was not convincing (29). Our study demonstrated additional social factors that would contribute to the differential risk between genders. We propose that differential access to an in-home caregiver and the wealthy are important contributing factors for mortality risk among frail older persons.

Several studies have examined the interaction of SES and frailty on mortality (31–33) and reported that higher SES can attenuate the mortality risk only in fit older adults (31, 32), a finding that is consistent with our study. Moreover, previous longitudinal studies exploring gender effects and clinical outcomes in older adults with different SES have reported mixed results (34–37). Some studies demonstrated that SES had strong benefits on health outcomes in older men, but the benefits were attenuated in women (34, 35). Other studies have reported no gender influence on SES and disability-free life expectancy, especially in countries with high expenditures on elder care (36, 37). Our study demonstrates the different effects of SES on mortality between older men and women with different frailty severity in resource-limited settings. We hypothesized that cultural norms on gender roles may partially explain the influence of gender on the relationship between SES and mortality in frail older persons. Since women are traditionally assigned to be the main housekeeper for families, these responsibilities may reduce the opportunity for consistent self-care among non-frail women, reducing the benefit of higher SES on their health, especially at lower levels of wealth. If this is the case for lower- and middle-income countries, it suggests a window of opportunity for interventions that would benefit a substantial proportion of pre-frail older women. Given the evidence of benefit from interventions for community-dwelling pre-frail older people (38), appropriate programs targeted toward older women should be considered. This strategy might be one path to mitigate gender inequity by ensuring access to health and social care services.

We found that having a caretaker reduced mortality risk for both genders, but at different magnitudes, with the most dramatic effect observed among men with severe frailty. Previous studies indicated that the concept of frailty was not well-understood or well-accepted among older people (39). It is possible that older people with frailty are not aware of the support they require, and they may decline to accept help when it is offered. The results suggest that the availability of caretakers could support seniors’ health. A previous study reported that social isolation has a negative impact on health, especially in frail older adults (40). A recent systematic review also demonstrated that the social vulnerability index, including the situation of living alone, has a significant effect on survival (41). The positive effect of a caretaker on a senior’s health could be due to physical support such as assistance for seeking medical care, medication management, and also mental support for loneliness and social security (42).

In our study, despite being severely frail, a substantial proportion of older people reported having no caretaker and was therefore at increased risk of mortality. This finding has practical applications for the national policy on health and social services for frail older people that require complex care and underscore the need to educate the wider society on issues related to aging and frailty.

Finally, we observed that chronological age is not a reliable predictor of mortality among fit older persons, a result that is consistent with several studies (2, 43, 44). Biological age as measured by cumulative deficits conceptualized in the frailty index is a more useful index for health and well-being than chronological age. Interventions should be prioritized for frail and pre-frail older people to reverse frailty status and prevent adverse clinical outcomes (38).

Our study has strengths and limitations. We developed the frailty index from a large, representative national cohort in Thailand with long-term follow-up. The proposed cutoff points for pre-frailty and frailty used recommended methods that have been validated in other population-based cohorts. This modified version of the TFI reliably predicts mortality in several viewpoints of analysis conducted. The additional proposed explanation for the association between social factors, frailty status, and intervention might be applicable in resource-limited settings. Nevertheless, further research to explore the predictive ability for other clinical outcomes, such as healthcare utilization for different frailty statuses in resource-limited settings, would be valuable. The small number of participants in certain frailty categories may have reduced precision in some estimates. Another limitation was in relation to the nature of the cohort study where the affirmation of participants’ frailty status during the follow-up was not ascertained.

## Conclusion

We constructed a modified and more practical version of the TFI to classify frailty status in community-dwelling older people. We estimated different mortality risks by gender for each frailty status, economic status, and social support. We identified the possible modifiable factor where early identification and intervention for older people might reduce mortality risk and improve quality of life. Our findings can inform policy decisions to set priority for allocating services in resource-limited settings.

## Data availability statement

The original contributions presented in this study are included in this article/Supplementary material, further inquiries can be directed to the corresponding authors.

## Ethics statement

The studies involving human participants were reviewed and approved by Human Research Ethics Office, Faculty of Medicine Siriraj Hospital, Mahidol University, Bangkok, Thailand. The patients/participants provided their written informed consent to participate in this study.

## Author contributions

EM, VS, and WA: conceptualization. VS and WA: methodology. RP and VS: formal analysis. WA: resources and data curation. EM and VS: writing the original draft of the manuscript. EM, VS, SS, US-A, AS, and WA: review and editing of the manuscript. VS: supervision. WA: project administration. All authors have read and agreed with the submitted version of the manuscript.
