# Corrigendum: Severity of frailty using modified Thai Frailty Index, social factors, and prediction of mortality among community-dwelling older adults in a middle-income country

**DOI:** 10.3389/fmed.2023.1272092

**Published:** 2023-08-31

**Authors:** Ekkaphop Morkphrom, Varalak Srinonprasert, Unchana Sura-amonrattana, Arunotai Siriussawakul, Supawadee Sainimnuan, Rinrada Preedachitkun, Wichai Aekplakorn

**Affiliations:** ^1^Division of Geriatric Medicine, Department of Medicine, Faculty of Medicine Siriraj Hospital, Mahidol University, Bangkok, Thailand; ^2^Siriraj Geriatric Internal Medicine Research Group, Research Department, Faculty of Medicine Siriraj Hospital, Mahidol University, Bangkok, Thailand; ^3^Integrated Perioperative Geriatric Excellent Research Center, Faculty of Medicine Siriraj Hospital, Mahidol University, Bangkok, Thailand; ^4^Siriraj Health Policy Unit, Faculty of Medicine Siriraj Hospital, Mahidol University, Bangkok, Thailand; ^5^Department of Anesthesiology, Faculty of Medicine Siriraj Hospital, Mahidol University, Bangkok, Thailand; ^6^Department of Community Medicine, Faculty of Medicine Ramathibodi Hospital, Mahidol University, Bangkok, Thailand

**Keywords:** frailty, older, mortality risk, Thailand, caretaker

In the published article, an author name was incorrectly written as Unchana Sura-amonratana. The correct spelling is Unchana Sura-amonrattana.

The authors apologize for this error and state that this does not change the scientific conclusions of the article in any way. The original article has been updated.

