# Supplementary material for: Severity of frailty using modified Thai frailty index, social factors, and prediction of mortality among community-dwelling older adults in a middle-income country
Source: Front Med (Lausanne). 2022 Dec 9;9:1060990. doi: 10.3389/fmed.2022.1060990 (PMC9780471; doi:10.3389/fmed.2022.1060990)
Supplement: Supplementary file 1 [file Data_Sheet_1.PDF]

frontiers

Supplementary Material

Supplementary Table 1. Deficits included in the modified Thai frailty index (TFI).

Variables	Definition	Value
1. Hypertension	Systolic BP ≥ 140 mmHg or diastolic BP ≥ 90 mmHg or current use of anti-hypertensive medication.	Yes = 1, No= 0
2. Diabetes mellitus	Medical diagnosis of diabetes with current use of anti-diabetic medication within 2 weeks; or fasting plasma glucose ≥ 126 mg/dL.	Yes = 1, No= 0
3. Stroke	Self-report or medical diagnosis of stroke	Yes = 1, No= 0
4. COPD	Self-report or medical diagnosis of COPD	Yes = 1, No= 0
5. Chronic kidney disease	Estimated GFR < 60 ml/min/1.73m^2 for at least 3 months	Yes = 1, No= 0
6. History of falls	Self-report for history of falls in past 6 months	Yes = 1, No= 0
7. Dental problems	Have less than 20 teeth or use dentures	Yes = 1, No= 0
8. Hearing problems	Self-report of hearing impairment or use hearing aids	Yes = 1, No= 0
9. Low Body mass index	Body mass index < 18.5 kg/m^2	Yes = 1, No= 0
10. Poor health status	Self-rating their general health<br>"In general, how would you rate your health today?"<br>(very good, good, moderate, bad, very bad)	Moderate, bad or very bad = 1;<br>Good or very good = 0
11. Poor quality of life	Self-rating their overall quality of life<br>"Considering on your health, your ability to perform activities of daily living, your personal relationship and condition of your living place, how satisfied are you with your life as a whole these days?"<br>(very satisfied, satisfied, moderate, dissatisfied, very dissatisfied)	moderate, dissatisfied, or very dissatisfied = 1;<br>satisfied or very satisfied = 0
12. Depressed mood	Self-report of feeling sad, empty, or depressed during the last 12 months.	Yes = 1, No= 0
13. Fatigue	Self-report of feeling decreased energy or tired all the time during the last 12 months	Yes = 1, No= 0

Variables	Definition	Value
14. Sleep difficulty	Self-report of sleep problems such as falling asleep, waking up frequently during the night or waking up too early in the morning during the last 30 days	Yes = 1, No= 0
15. Loss of interest	Self-report of loss of interest in most things that usually enjoy such as personal relationships, work or hobbies/recreation during the last 12 months	Yes = 1, No= 0
16. Need assistance for bathing	Report of need assistance for bathing or washing whole body.	Yes = 1, No= 0
17. Need assistance for dressing	Report of need assistance for getting dressed (including buttoning or zipping up.)	Yes = 1, No= 0
18. Need assistance for eating	Report for need assistance with eating (including cutting up your food)	Yes = 1, No= 0
19. Need assistance for indoor walking	Report for need assistance for moving around inside their home.	Yes = 1, No= 0
20. Need assistance for toileting	Report of need assistance for getting to and using the toilet	Yes = 1, No= 0
21. Need assistance for transferring	Report of need assistance for getting up from lying down.	Yes = 1, No= 0
22. Urinary incontinence	Report of unintentional passing of urine during the last 24 hours	Yes = 1, No= 0
23. Fecal incontinence	Report of unintentional passing of stool during the last 7 days	Yes = 1, No= 0
24. Need assistance in drug management	Report of need assistance from caretaker to prepare medication.	Yes = 1, No= 0
25. Need assistance for doing housework	Report of need assistance to housework including sweeping the floor, tidying the bedroom.	Yes = 1, No= 0
26. Need assistance for upstairs walking	Report of inability to walk up 10-stepped stair without pausing or assistance.	Yes = 1, No= 0
27. Need help for walking more than 400 m.	Report of inability to walk 400 m. without pausing or assistance.	Yes = 1, No= 0
28. Reduced hand grip strength	Hand grip strength per 1 kg. of body weight $\leq$ 0.48 in men or $\leq$ 0.35 in women	Yes = 1, No= 0
29. Impaired calculation	Ask individuals to serially subtract seven from 100 for three sequences.	Unable to provide any correct answer= 1; $\geq$ one correct answer= 0

Variables	Definition	Value
30. Impaired memory	Ask individuals to remember list of three words (tree, car, hand). Three minutes later, ask them to recall as many words as they can.	Recall ≤ 1 word = 1,<br>Recall ≥ 2 words = 0

Supplementary Table 2. Baseline characteristics by frailty status among male participants.

Variable	Fit (n=1225)	Pre-frail (n=2275)	Mildly frail (n=486)	Severely Frail (n=32)	p-value
Age, mean±SD (year)	67.5±18.5	69.4±6.8	72.8±7.8	75.1±6.0	<0.001
Good overall health status, n (%)*	898 (71.6%)	738 (32.4%)	56 (11.5%)	0 (0.0%)	<0.001
History of smoking, n (%)	931 (74.2%)	1833 (80.6%)	406 (83.5%)	28 (87.5%)	<0.001
Wealth index quintile, n (%)					<0.001
1st	181 (14.4%)	450 (19.8%)	114 (23.5%)	12 (37.5%)	
2nd	183 (14.6%)	407 (17.9%)	89 (18.3%)	5 (15.6%)	
3rd	248 (19.8%)	461 (20.3%)	117 (24.1%)	4 (12.5%)	
4th	256 (20.4%)	441 (19.4%)	91 (18.7%)	7 (21.9%)	
5th	387 (30.8%)	516 (22.7%)	75 (15.4%)	4 (12.5%)	
BMI, mean±SD (kg/m^2)	22.6±3.3	22.6±4.0	22.3±4.4	20.9±4.2	<0.001
Gait speed, median [IQR] (m/s)	0.84 (0.71,0.96)	0.76 (0.64,0.90)	0.65 (0.52,0.78)	0.37 (0.28,0.56)	<0.001
Hand grip, median [IQR] (kg)	31.00 (27.90,36.00)	28.60 (24.10,33.09)	24.90 (20.47,29.32)	21.29 (17.02,24.90)	<0.001
History of falls in 6 months, n (%)	70 (5.6%)	343 (15.1%)	132 (27.2%)	14 (43.8%)	<0.001
Number of BADL deficit**, median [IQR]	1 [1,2]	2 [1,2]	2 [1,2]	2 [2,4]	<0.001
Number of IADL deficit***, Median [IQR]	1 [1,1]	1 [1,2]	2 [1,3]	4 [3,5]	<0.001
Number of comorbidities, median [IQR]	0 [0, 1]	1 [0, 2]	2 [1, 3]	3 [2,4]	<0.001
Living with caretaker, n (%)	344 (27.5%)	791 (35.0%)	249 (51.7%)	25 (78.1%)	<0.001
Depressive mood, n (%)	31 (2.5%)	354 (15.6%)	237 (48.8%)	20 (62.5%)	<0.001
Cognitive impairment, n (%)	40 (3.2%)	199 (8.7%)	79 (16.3%)	12 (37.5%)	<0.001
Hypertension, n (%)	170 (13.5%)	773 (34.0%)	257 (52.9%)	18 (56.3%)	<0.001
Diabetes, n (%)	74 (5.9 %)	388 (17.1%)	121 (24.9%)	12 (37.5%)	<0.001
Stroke, n (%)	11 (0.9%)	72 (3.2%)	52 (10.7%)	12 (37.5%)	<0.001
Chronic obstructive pulmonary disease, n (%)	9 (0.7%)	93 (4.1%)	35 (7.2%)	5 (15.6%)	<0.001
Death, 1000 person-years (95%CI)	19.9 (16.9-23.4)	32.5 (29.5-35.7)	64.8 (55.9-75.2)	164.3 (112.1 -240.8)	<0.001

** Overall health status is self-report overall heath from the question "In general, how would you rate your health today?" (very good, good, moderate, bad, very bad).*

*** Basic activity of daily living (BADL) deficit means inability to do one of the following by themselves: bathing, dressing, eating, transferring from bed to chair, indoor ambulation, stair climbing, toileting, urinary continence, and fecal continence.*

**** Instrumental activity of daily living (IADL) deficit means inability to do one of the following by themselves: paying for bill, medication use, light housework (e.g., sweeping floor, tidying room), heavy housework (e.g., mobbing floor, carrying water bucket), public transportation, telephone use.*

IQR means interquartile range that represent the range from 25th percentile to 75th percentile of data

Supplementary Table 3. Baseline characteristics by frailty status among female participants.

Variable	Fit (n=1915)	Prefrail (n=2387)	Mildly frail (n=780)	Severely Frail (n=65)	p-value
Age, mean±SD (year)	67.3±20.6	68.9±6.7	71.9±7.5	76.0±7.3	<0.001
Good overall health status, n (%)*	634 (69.3%)	834 (34.9%)	105 (13.5%)	2 (3.1%)	<0.001
History of smoking, n (%)	158 (17.3%)	609 (25.5%)	249 (31.9%)	21 (32.3%)	<0.001
Wealth index quintile, n (%)					<0.001
1st	160 (17.5%)	500 (20.9%)	206 (26.4%)	11 (16.9%)	
2nd	134 (14.6%)	412 (17.3%)	140 (17.9%)	18 (27.7%)	
3rd	167 (18.3%)	483 (20.2%)	172 (22.1%)	15 (23.1%)	
4th	163 (17.8%)	481 (20.2%)	124 (15.9%)	11 (16.9%)	
5th	291 (31.8%)	511 (21.4%)	138 (17.7%)	10 (15.4%)	
BMI, mean±SD (kg/m^2)	23.5±3.8	24.0±4.8	24.0±5.1	22.8±4.0	<0.001
Gait speed, median [IQR] (m/s)	0.74 (0.62,0.86)	0.68 (0.56,0.80)	0.58 (0.46,0.69)	0.48 (0.36,0.58)	<0.001
Hand grip, median [IQR] (kg)	21.60 (18.50,24.50)	19.90 (16.70,22.79)	17.70 (14.70,20.79)	16.10 (12.90,18.10)	<0.001
History of falls in 6 months, n (%)	64 (7.0%)	510 (21.4%)	294 (37.7%)	31 (47.7%)	<0.001
Number of BADL deficit**, median [IQR]	1 [1,2]	2 [1,2]	2 [1,2]	2 [2,3]	<0.001
Number of IADL deficit***, Median [IQR]	1 [1,1]	1 [1,2]	2 [1,3]	3 [2,4]	<0.001
Number of comorbidities, median [IQR]	0 [0, 1]	1 [0, 2]	2 [1,3]	2 [2, 3]	<0.001
Living with caretaker, n (%)	263 (28.8%)	880 (37.0%)	358 (46.1%)	39 (60.0%)	
Depressive mood, n (%)	20 (2.2%)	438 (18.3%)	393 (50.4%)	57 (87.8%)	<0.001
Cognitive impairment, n (%)	54 (5.9%)	245 (10.3%)	194 (24.9%)	23 (35.4%)	<0.001
Hypertension, n (%)	129 (14.1%)	900 (37.7%)	443 (56.8%)	46 (70.8%)	<0.001
Diabetes, n (%)	61 (6.7%)	434 (18.2%)	223 (28.6%)	22 (33.8%)	<0.001
Stroke, n (%)	3 (0.3%)	47 (5.8%)	45 (5.8%)	9 (13.8%)	<0.001
Chronic obstructive pulmonary disease, n (%)	1 (0.1%)	24 (1.0%)	17 (2.2%)	3 (4.6%)	<0.001
Death, 1000 person-years (95%CI)	8.8	22.3	32.4	73.7	<0.001

	(6.6-11.6)	(20.0-24.9)	(27.6 -38.1)	(50.5 -107.5)

** Overall health status is self-report overall heath from the question "In general, how would you rate your health today?" (very good, good, moderate, bad, very bad).*

*** Basic activity of daily living (BADL) deficit means inability to do one of the following by themselves: bathing, dressing, eating, transferring from bed to chair, indoor ambulation, stair climbing, toileting, urinary continence, and fecal continence.*

**** Instrumental activity of daily living (IADL) deficit means inability to do one of the following by themselves: paying for bill, medication use, light housework (e.g., sweeping floor, tidying room), heavy housework (e.g., mobbing floor, carrying water bucket), public transportation, telephone use.*

IQR means interquartile range that represent the range from 25th percentile to 75th percentile of data
